# Differential effects of antipsychotic drugs on contrast response functions of retinal ganglion cells in wild-type Sprague-Dawley rats and P23H retinitis pigmentosa rats

**DOI:** 10.1371/journal.pone.0218200

**Published:** 2019-06-10

**Authors:** Ralph Jensen

**Affiliations:** Research Service, VA Boston Healthcare System, Boston, Massachusetts, United States of America; University of Florida, UNITED STATES

## Abstract

Antipsychotic drugs haloperidol and clozapine have been reported to increase the sensitivity of retinal ganglion cells (RGCs) to flashes of light in the P23H rat model of retinitis pigmentosa. In order to better understand the effects of these antipsychotic drugs on the visual responses of P23H rat RGCs, I examined the responses of RGCs to a drifting sinusoidal grating of various contrasts. In-vitro multielectrode array recordings were made from P23H rat RGCs and healthy Sprague-Dawley (SD) rat RGCs. Retinas were stimulated with a drifting sinusoidal grating with eight values of contrast (0, 4, 6, 8.5, 13, 26, 51, and 83%). Contrast response functions based on response amplitudes were fitted with a hyperbolic ratio function and contrast thresholds were determined from the fitted curves. SD rat RGCs were divided into two categories, saturating and non-saturating cells, based on whether they showed saturation of responses at high contrast levels. Most SD rat RGCs (58%) were saturating cells. Haloperidol and clozapine decreased the responses of saturating SD rat RGCs to all grating contrasts, except for the highest contrast tested. Clozapine also decreased the responses of non-saturating SD rat RGCs to all grating contrasts, except for the highest contrast tested. Haloperidol did not however significantly affect the responses of non-saturating SD rat RGCs. Haloperidol and clozapine increased the contrast thresholds of both saturating and non-saturating cells in SD rat retinas. Most (73%) P23H rat RGCs could be categorized as either saturating or non-saturating cells. The remaining ‘uncategorized’ cells were poorly responsive to the drifting grating and were analyzed separately. Haloperidol and clozapine increased the responses of non-saturating and uncategorized P23H rat RGCs to most grating contrasts, including the highest contrast tested. Haloperidol and clozapine also increased the responses of saturating P23H rat RGCs to most grating contrasts but these increases were not statistically significant. Haloperidol and clozapine decreased the contrast thresholds of saturating cells, non-saturating cells and uncategorized cells in P23H rat retinas, although the decrease in contrast thresholds of saturating cells was not found to be statistically significant. Overall, the findings show that haloperidol and clozapine have differential effects on the contrast response functions of SD and P23H rat RGCs. In contrast to the effects observed on SD rat RGCs, both haloperidol and clozapine increased the responsiveness of P23H rat RGCs to both low and high contrast visual stimuli and decreased contrast thresholds.

## Introduction

Retinitis pigmentosa (RP) is an inherited retinal degenerative disease in which there is a progressive loss of rod photoreceptors, followed by a loss of cone photoreceptors. This genetically heterogeneous disease affects about 1 in 4000 people worldwide [1]. Unfortunately, no approved pharmacological treatment exists for patients with RP. However, studies conducted in animal models of RP have shown multiple pharmacological agents that can slow down photoreceptor degeneration. These neuroprotectants included nerve growth factor [2], fluocinolone acetonide [3, 4], 9-cis-retinyl acetate [5, 6], and N-acetylcysteine [7], all of which have moved into clinical trials with RP subjects (clinicaltrials.gov; clinicaltrialsregister.eu).

With the loss of photoreceptors in RP and age-related macular degeneration (AMD) there is remodeling of cells of the inner retina, including horizontal cells, bipolar cells and amacrine cells [8, 9]. Not surprisingly, this remodeling affects the activity of RGCs. One of the earliest documented findings is a change in the spontaneous spike activity of RGCs in the rd1 mouse (a model of RP with a mutation in the *Pde6b* gene), including the appearance of oscillatory bursts of spikes [10, 11]. Another animal model of RP is the P23H rat, which was created by the incorporation of a mutated mouse rhodopsin gene in the Sprague-Dawley rat. In the P23H rat retina, a shrinkage in the receptive field size of RGCs [12] and an abnormally long-latency ON response in a subpopulation of RGCs [13] have been reported.

Whereas neuroprotectants aim to preserve vision by slowing down or halting the neurodegenerative processes in photoreceptors, pharmacological agents that target cellular signaling in the inner retina have the potential to restore some visual function. Indeed, blocking either GABA_C_ or mGlu1 receptors in the P23H rat retina increases the responses of RGCs to flashes of light [14, 15] and decreases contrast thresholds to a drifting sinusoidal grating [16]. The antipsychotic drugs haloperidol and clozapine have also been shown to increase the responses of P23H rat RGCs to flashes of light, possibly due to the actions of haloperidol and clozapine on dopamine D2 receptors and serotonin 5-HT2A receptors, respectively [17]. The present study was undertaken to determine how haloperidol and clozapine affect the response amplitudes and contrast thresholds of RGCs in P23H and SD rat retinas to a drifting sinusoidal grating – a visual stimulus that is commonly used to measure contrast sensitivity behaviorally in rodents.

## Materials and methods

### Animals

P23H-line 3 homozygous rats and Sprague-Dawley (SD) rats of 50–57 weeks of age were used in this study. Breeding pairs of P23H-line 3 [SD-Tg(P23H)3Lav] homozygous rats were obtained from the Rat Resource and Research Center (Columbia, MO). SD rats were obtained from Harlan Laboratories (Indianapolis, IN). Rats were housed in a room that was kept on a 12 hr light/dark cycle using standard fluorescent lighting. During the light cycle, the illumination at the level of the cages was 100–200 lux. This study was carried out in accordance with the recommendations in the Guide for the Care and Use of Laboratory Animals of the National Institutes of Health. The protocol was approved by the VA Boston Healthcare System Committee on Use and Care of Animals (Protocol Number: 348-J-060517). All efforts were made to minimize animal stress.

### Extracellular recordings

As described in detail previously [16], following euthanasia of a rat with sodium pentobarbital, an eye was removed and hemisected. After removal of the vitreous, the eyecup was submerged in carboxygenated Ames' Medium (supplemented with 2 g/L sodium bicarbonate and 1.5 g/L d-glucose). A square piece of retina measuring ∼2–3 mm on each side was cut out and placed with the ganglion cell side down onto a 64-channel planar Muse MEA (Axion Biosystems Inc.). A gravity-flow system administered the carboxygenated Ames' Medium to the retina at a flow rate of 1.5 ml min^−1^ and temperature of 31 to 33°C. The retina was superfused for at least 20 min before data acquisition.

Electrode recordings were digitized at 20 kHz and stored on a hard disk for offline analysis. Detection of single action potentials (spikes) was performed using Axion Biosystem software and setting a voltage threshold 5–6 fold the standard deviation of the noise over 200 Hz high-pass filtered traces. Principal component analysis of the spike waveforms was used for sorting spikes generated by individual cells (Offline Sorter, Plexon).

### Visual stimulation

Visual stimuli, generated with the PsychoPy (v1.81) package [18], were delivered to a DLP projector. Images from the projector were minified with external lenses and focused onto the photoreceptor surface of the retina with a 10X microscope objective. Visual stimuli consisted of drifting sinusoidal gratings that were presented with a mean illuminance that equaled that of the background. The mean stimulus illuminance was adjusted by neutral density filters positioned adjacent to the projector output. The mean stimulus illuminance was set at 15 lux in experiments conducted on SD rat retinas but varied from 15 to 60 lux in experiments conducted on P23H rat retinas. Spatial frequency of the sinusoidal gratings was held constant at 1 cycle/mm, and temporal frequency was held constant at 2 cycles/s. All gratings were presented within a circular patch of 2.4 mm diameter, centered over the MEA. Retinas were stimulated with eight values of contrast (0, 4, 6, 8.5, 13, 26, 51, and 83%). Contrast was defined by the Michelson formula, 100% x (Lmax−L_min_)/ (L_max_ + L_min_), where L_max_ and L_min_ are the maximum and minimum illuminance levels of the sinusoidal grating. At each grating contrast, seven trials were presented. Each trial started with a 4 s presentation of a uniform field (same mean illuminance as the test grating) followed by a 6 s presentation of a drifting sinusoidal grating. An interval of 20 s between trials was chosen to minimize possible effects of stimulation history.

### Drugs

Haloperidol and clozapine (both from Tocris Bioscience) were added to the bath at 0.5 μM and 5 μM, respectively, using a syringe pump as described previously [19]. The effects of a drug on RGC light responses were examined only after the drug had been bath applied to the retinal preparation for ~10 min to ensure stable responses.

### Data analysis

Spikes from RGCs were imported into Neuroexplorer software (Nex Technologies) to create post-stimulus time histograms (PSTHs) with a 10 ms bin width, averaged across 7 repetitions of the same contrast. Each histogram was Fourier transformed with OriginPro10 software (OriginLab Corp.) to obtain the amplitude of the fundamental stimulus frequency (F1). The response amplitude of each cell was obtained by subtracting the baseline (F1 amplitude) response determined with 0% grating contrast from the F1 amplitude obtained at each contrast level. Group comparisons of response amplitudes to various grating contrasts between drug-treated and control (pre-drug tested) were conducted with a two-tailed paired Student’s t-test (OriginPro10 software). P values were corrected for multiple comparisons using the Holm-Bonferroni method. Holm-corrected P values < 0.05 were deemed significantly different. Response amplitudes of each cell were also used to construct a contrast response function, which was fitted with the hyperbolic ratio function [20]. Contrast thresholds of cells were determined from the contrast response functions. Medians are used to report contrast threshold data since for some cells the contrast threshold value was immeasurable (i.e., exceeded the highest contrast stimulus tested). Group comparisons of contrast thresholds were conducted with the Wilcoxon signed-rank test. P values < 0.05 were considered statistically significant.

## Results

**Fig 1:**
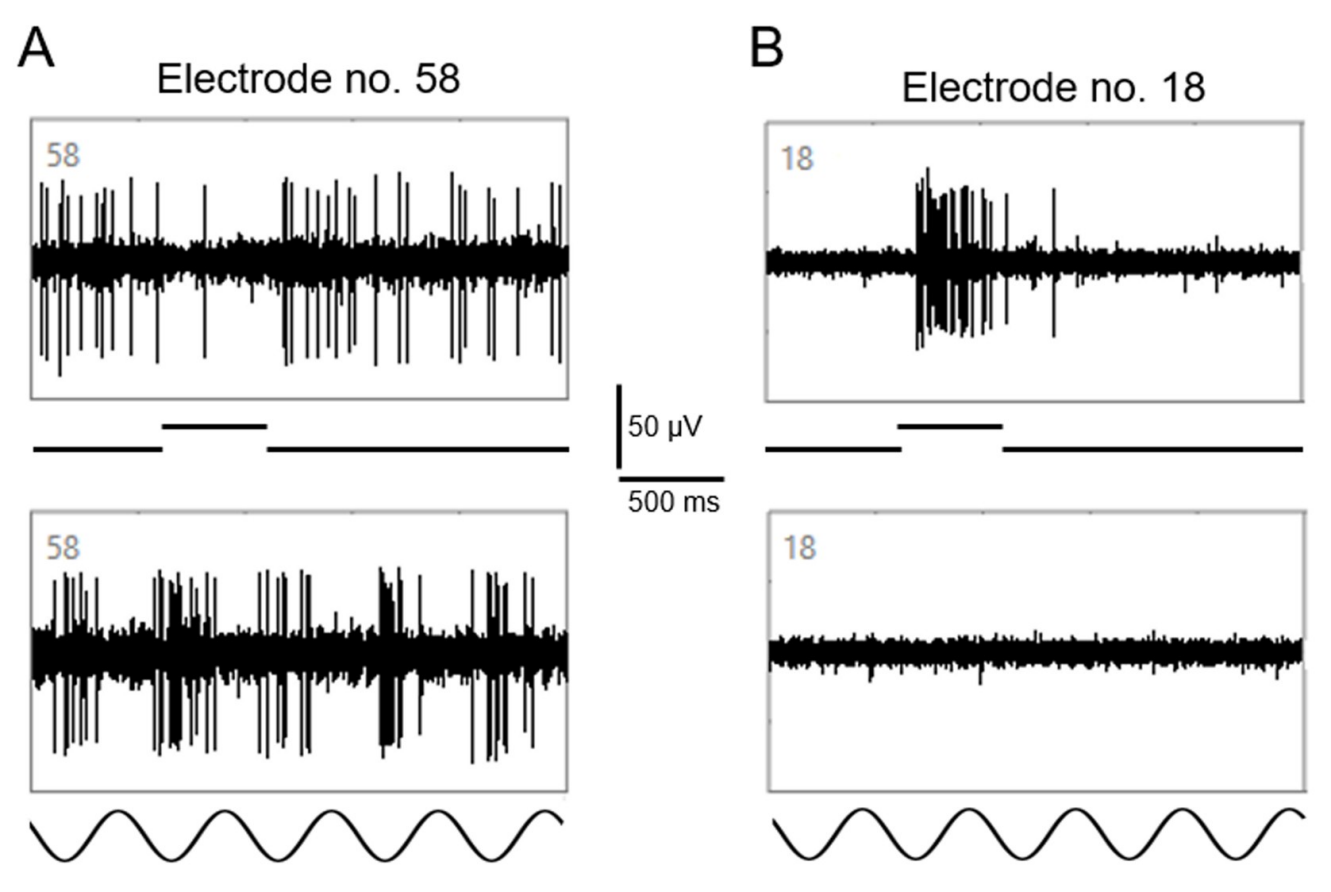
MEA recordings from two RGCs. Upper traces in (A) and (B) show the responses of two RGCs to a full-field flash (500 ms) of light. Cell recorded by electrode 58 was an OFF cell; cell recorded by electrode 18 was an ON cell. Lower traces in (A) and (B) show the responses of the two RGCs to the drifting sinusoidal grating. Whereas the cell recorded by electrode 58 clearly responded to the drifting grating, no response was elicited from the cell recorded by electrode 18.

Spike activity of many SD and P23H rat RGCs was modulated by the full-field drifting sinusoidal grating (spatial frequency: 1 cycle/mm, temporal frequency: 2 cycles/s). However, about one-third of the recorded RGCs (n = 415) were unresponsive to this grating in both the presence and absence of an antipsychotic drug. Fig 1 shows recordings from two RGCs (from one retina) to the drifting sinusoidal grating. Both cells elicited a response to a full-field flash of light (upper traces in A and B) but only one RGC responded to the drifting sinusoidal grating (lower trace in panel A). Cells that did not show a response to the drifting sinusoidal grating in both the presence and absence of an antipsychotic drug were not included in the data analyses. As will be shown below, the responses of SD and P23H rat RGCs to the drifting grating were affected differently by the antipsychotic drugs haloperidol and clozapine. Results from SD rat RGCs will be described first.

### Effects of antipsychotic drugs on contrast response functions of SD rat RGCs

**Fig 2:**
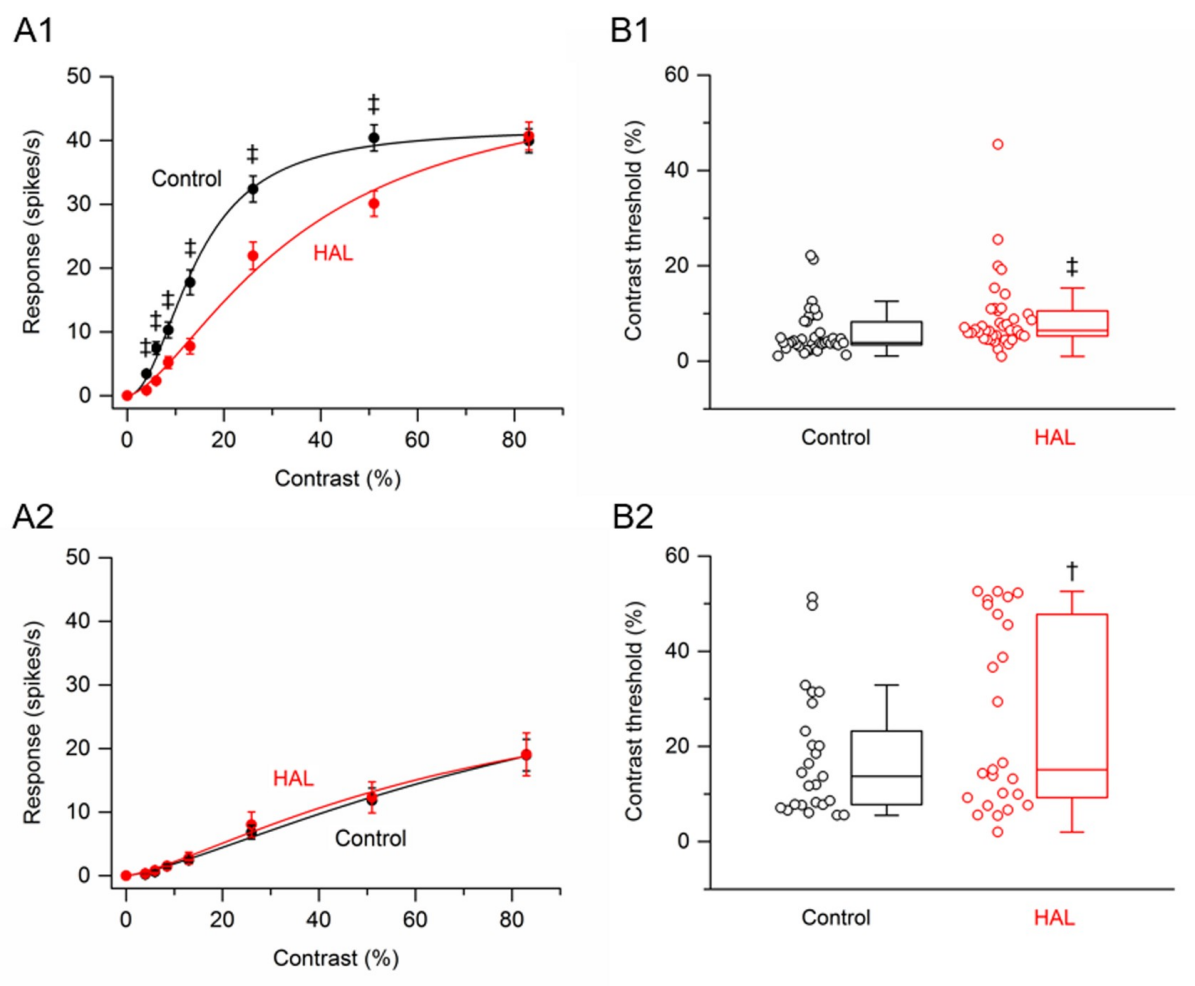
Effects of haloperidol on responses of SD rat RGCs to drifting sinusoidal grating of various contrasts. (A1 –A2) RGC contrast response functions. (A1) Contrast response function from saturating RGCs (n = 39) before and after application of haloperidol. (A2) Contrast response function from non-saturating RGCs (n = 25) before and after application of haloperidol. Data points in (A1) and (A2) are the mean ± SEM (errors smaller than the symbol size are not visible). ‡ P < 0.001 (Holm-Bonferroni multiple correction). (B1 –B2) RGC contrast thresholds. (B1) Contrast thresholds for saturating RGCs (n = 39) before and after application of haloperidol. (B2) Contrast thresholds for non-saturating RGCs (n = 25) before and after application of haloperidol. In (B1) and (B2), boxes represent the interquartile range (IQR) between first and third quartiles and the line inside represents the median. Whiskers denote the lowest and highest values within 1.5 x IQR from the first and third quartiles. Circles represent all data points. † P < 0.01, ‡ P < 0.001 (Wilcoxon signed-rank test).

Data were obtained from 64 RGCs (4 retinas, n = 10–20 cells per retina) that were treated with the antipsychotic drug haloperidol. Based on the fit of the hyperbolic ratio function (see Materials and methods), RGCs were arbitrarily subdivided into two populations: saturating and non-saturating cells. As defined previously [16], saturating RGCs included those cells whose value at 83% contrast was within 10% of the calculated plateau value; all other cells were categorized as non-saturating RGCs. Of the cells treated with haloperidol, 39 cells (61%) were saturating RGCs and 25 cells (39%) were non-saturating RGCs. Fig 2(A1) shows the contrast response function averaged from the saturating RGCs before and after application of haloperidol. Haloperidol significantly reduced the response amplitudes to all grating contrasts, except for the highest contrast (83%) tested. The greatest percentage reduction, 75%, occurred at 4% contrast. At higher contrasts (6% to 51%), haloperidol reduced the response amplitudes on average by 25–69%. Fig 2(A2) shows the contrast response function averaged from non-saturating RGCs before and after application of haloperidol. Haloperidol had no significant effect on the response amplitude at any grating contrast.

From the fitted hyperbolic ratio function, the contrast threshold of each cell could be determined. Contrast threshold was taken as a response amplitude of 2 spikes/s [16]. Box plots in (Fig 2B1 and 2B2) summarize the effects of haloperidol on the contrast thresholds of saturating and non-saturating RGCs. For the population of saturating RGCs, the median contrast thresholds were 3.90% before application of haloperidol and 6.44% after application of haloperidol. The difference between the medians was statistically significant (P < 0.001). For the population of non-saturating RGCs, the median contrast thresholds were 13.7% before application of haloperidol and 15.1% after application of haloperidol. Although the difference between the medians was small, it was statistically significant (P = 0.007). Thus, for both saturating and non-saturating RGCs, haloperidol increased contrast threshold.

**Fig 3:**
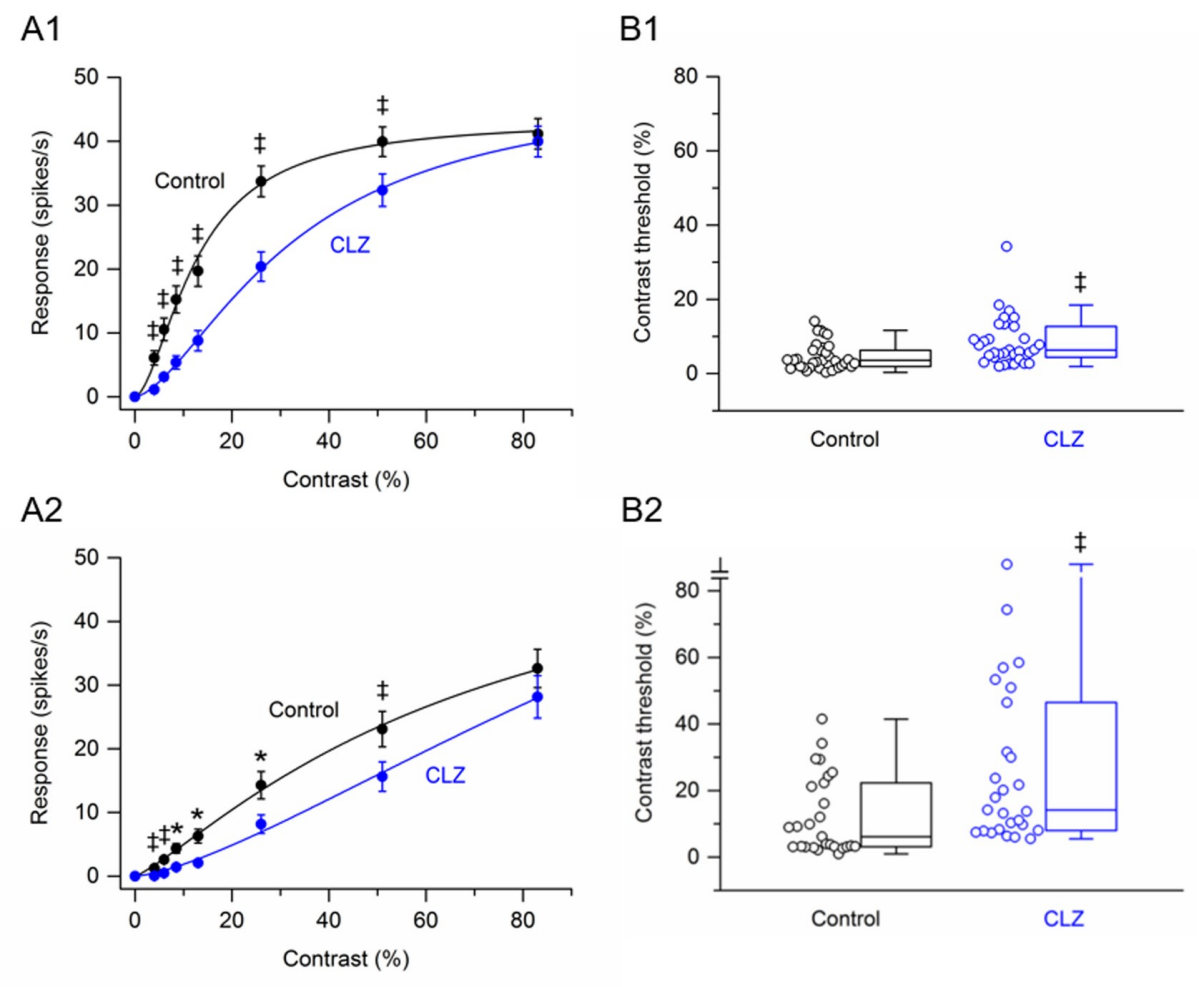
Effects of clozapine on responses of SD rat RGCs to drifting sinusoidal grating of various contrasts. (A1 –A2) RGC contrast response functions. (A1) Contrast response function from saturating RGCs (n = 33) before and after application of clozapine. (A2) Contrast response function from non-saturating RGCs (n = 27) before and after application of clozapine. Data points in (A1) and (A2) are the mean ± SEM. * P < 0.05, ‡ P < 0.001 (Holm-Bonferroni multiple correction). (B1 –B2) RGC contrast thresholds. (B1) Contrast thresholds for saturating RGCs (n = 33) before and after application of clozapine. (B2) Contrast thresholds for non-saturating RGCs (n = 27) before and after application of clozapine. In (B1) and (B2), boxes represent the interquartile range (IQR) between first and third quartiles and the line inside represents the median. Whiskers denote the lowest and highest values within 1.5 x IQR from the first and third quartiles. Circles represent all data points. Note the contrast threshold value for one cell in (B2) was immeasurable (i.e., exceeded 83%). ‡ P < 0.001 (Wilcoxon signed-rank test).

Data were obtained from 60 RGCs (4 retinas, n = 16–20 cells per retina) that were treated with the antipsychotic drug clozapine. Of the cells treated with clozapine, 33 cells (55%) were saturating RGCs. Fig 3(A1) shows the contrast response function averaged from saturating RGCs before and after application of clozapine. Like haloperidol, clozapine significantly reduced the response amplitudes to all grating contrasts, except for the highest contrast (83%) tested. The greatest percentage reduction, 82%, occurred at 4% contrast. At higher contrasts (6% to 51%), clozapine reduced the response amplitudes on average by 19–70%. Of the cells treated with clozapine, 27 cells (45%) were non-saturating RGCs. Fig 3(A2) shows the contrast response function averaged from non-saturating RGCs before and after application of clozapine. Whereas haloperidol had no significant effect on the response amplitude of non-saturating RGCs at any grating contrast, clozapine significant reduced the response amplitudes to all grating contrasts, except for the highest contrast (83%) tested. The greatest percentage reduction, 95%, occurred at 4% contrast. At higher contrasts (6% to 51%), clozapine reduced the response amplitudes on average by 32–81%.

Box plots in (Fig 3B1 and 3B2) summarize the effects of clozapine on the contrast thresholds of saturating and non-saturating RGCs. For the population of saturating RGCs, the median contrast thresholds were 3.57% before application of clozapine and 6.35% after application of clozapine. The difference between the medians was statistically significant (P < 0.001). For the population of non-saturating RGCs, the median contrast thresholds were 6.13% before application of clozapine and 14.2% after application of clozapine. The difference between the medians was statistically significant (P < 0.001). Thus, for both saturating and non-saturating RGCs, clozapine increased contrast threshold.

### Effects of antipsychotic drugs on contrast response functions of P23H rat RGCs

In the above experiments with SD rats, data were collected from retinas with the mean illuminance of the grating set at 15 lux. In experiments with P23H rats, the mean illuminance of the grating was initially set at 15 lux and the retina stimulated with a high contrast (83%) grating. If no RGC on the multielectrode array exhibited modulation of spike activity to the drifting grating, then the mean illuminance was increased 2-fold to 30 lux. If still no RGC showed any response modulation at this mean illuminance, then the mean illuminance was again increased 2-fold to 60 lux. In the event that no RGC responded at this mean illuminance, the retina was discarded and no data were collected. Data were collected from 5 retinas with the mean illuminance of the grating set at 15 lux, 2 retinas with the mean illuminance set at 30 lux, and 3 retinas with the mean illuminance set at 60 lux. In all retinas, there were RGCs that responded only to the highest grating contrast or two highest grating contrasts. Due to the insufficient number of data points, the fit quality of the hyperbolic ratio function was poor. These RGCs, henceforth referred to as ‘uncategorized’ cells, are analyzed separately from the other (saturating and non-saturating) cells.

**Fig 4:**
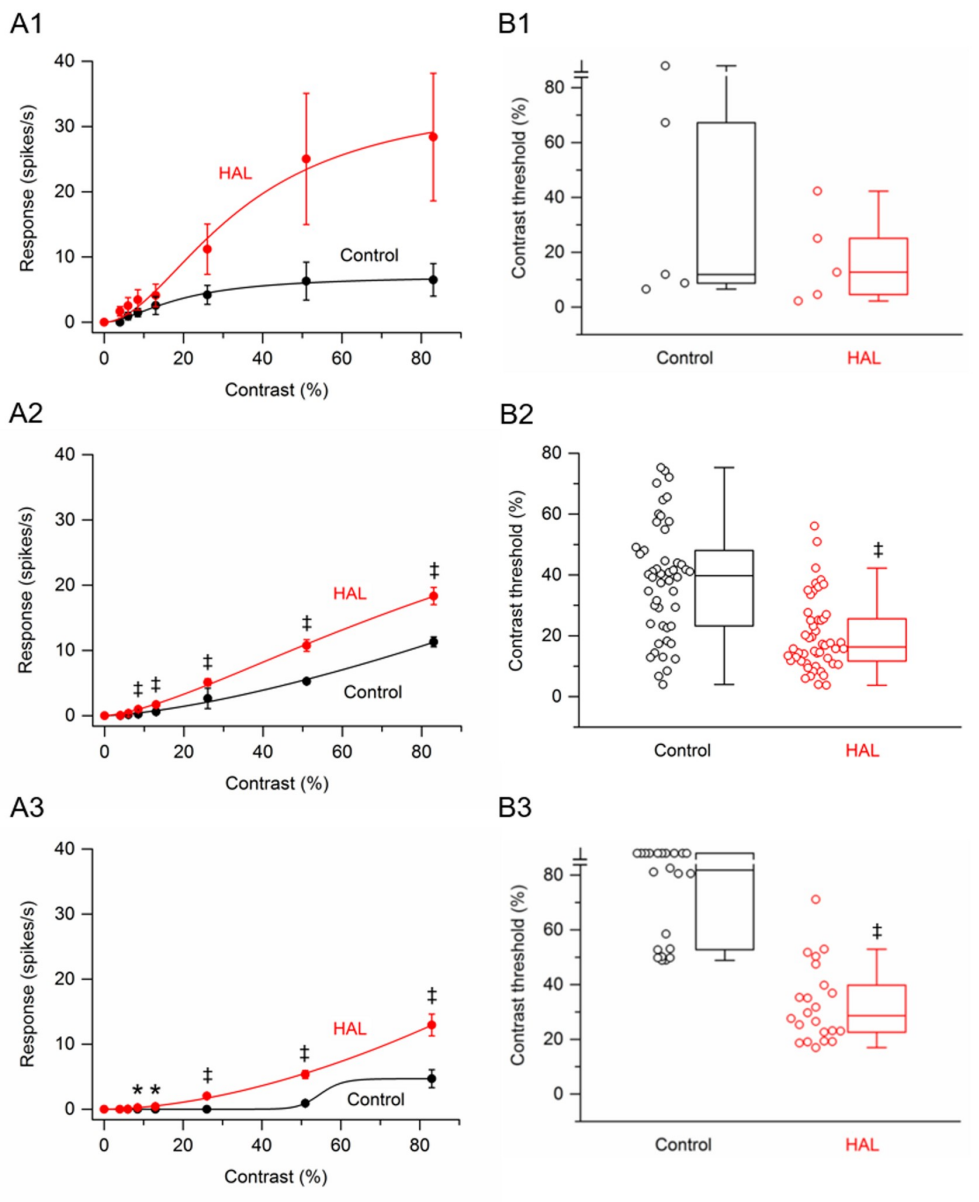
Effects of haloperidol on responses of P23H rat RGCs to drifting sinusoidal grating of various contrasts. (A1 –A3) RGC contrast response functions. (A1) Contrast response function from saturating RGCs (n = 5) before and after application of haloperidol. (A2) Contrast response function from non-saturating RGCs (n = 50) before and after application of haloperidol. (A3) Contrast response function from uncategorized RGCs (n = 22) before and after application of haloperidol. Data points in (A1), (A2) and (A3) are the mean ± SEM. * P < 0.05, ‡ P < 0.001 (Holm-Bonferroni multiple correction). Note that the control curve in (A3) is a bad fit, giving the impression that the cells were ‘saturating’ cells. This bad fit is because only two data points have values greater than zero. (B1 –B3) RGC contrast thresholds. (B1) Contrast thresholds for saturating RGCs (n = 5) before and after application of haloperidol. (B2) Contrast thresholds for non-saturating RGCs (n = 50) before and after application of haloperidol. (B3) Contrast thresholds for uncategorized RGCs (n = 22) before and after application of haloperidol. In (B1), (B2) and (B3), boxes represent the interquartile range (IQR) between first and third quartiles and the line inside represents the median. Whiskers denote the lowest and highest values within 1.5 x IQR from the first and third quartiles. Circles represent all data points. ‡ P < 0.001 (Wilcoxon signed-rank test). Note the contrast threshold values for one cell in (B1) and ten cells in (B3) were immeasurable (i.e., exceeded 83%).

Data were obtained from 77 RGCs (3 retinas at 15 lux, n = 56 cells; 1 retina at 30 lux, n = 10 cells; 1 retina at 60 lux, n = 11 cells) that were treated with the antipsychotic drug haloperidol. Of these RGCs, only 5 cells were saturating cells. The low percentage of saturating cells is not surprising since a similar finding has previously been reported in P23H-line 1 homozygous rat retinas [16]. Fig 4(A1) shows the contrast response function averaged from these saturating RGCs before and after application of haloperidol. Although the response amplitudes from these cells were on average larger at all grating contrasts in the presence of haloperidol, these increases failed to reach statistical significance. Fig 4(A2) shows the contrast response function averaged from non-saturating RGCs (n = 50) before and after application of haloperidol. Haloperidol significantly increased the response amplitudes to gratings of contrast from 8.5% to 83%. The greatest percentage increase, 322%, occurred at 8.5% contrast. At higher contrasts (13% to 83%), haloperidol increased the response amplitudes on average by 62–178%. Fig 4(A3) shows the contrast response function averaged from uncategorized RGCs (n = 22) before and after application of haloperidol. Before application of haloperidol, no responses were observed from any cell until the contrast reached 53%. In the presence of haloperidol, 1 cell responded to 6% contrast, 5 cells responded to 8.5% contrast, 7 cells responded to 13% contrast, and 19 cells responded to 26% contrast. Statistically, haloperidol significantly increased the response amplitudes to gratings of contrast from 8.5% to 83%.

Contrast thresholds were determined for saturating, non-saturating, and uncategorized RGCs. The data are displayed as three box plots in (Fig 4B1, 4B2 and 4B3). For the population of saturating RGCs, the median contrast thresholds were 11.9% before application of haloperidol and 12.7% after application of haloperidol. The difference between the medians was not statistically significant (P = 0.125). For the population of non-saturating RGCs, the median contrast thresholds were 39.7% before application of haloperidol and 16.3% after application of haloperidol. The difference between the medians was statistically significant (P < 0.001). For the population of uncategorized RGCs, the median contrast thresholds were 81.9% before application of haloperidol and 28.6% after application of haloperidol. The difference between the medians was statistically significant (P < 0.001). Taken together, haloperidol tended to decrease the contrast thresholds of P23H rat RGCs.

**Fig 5:**
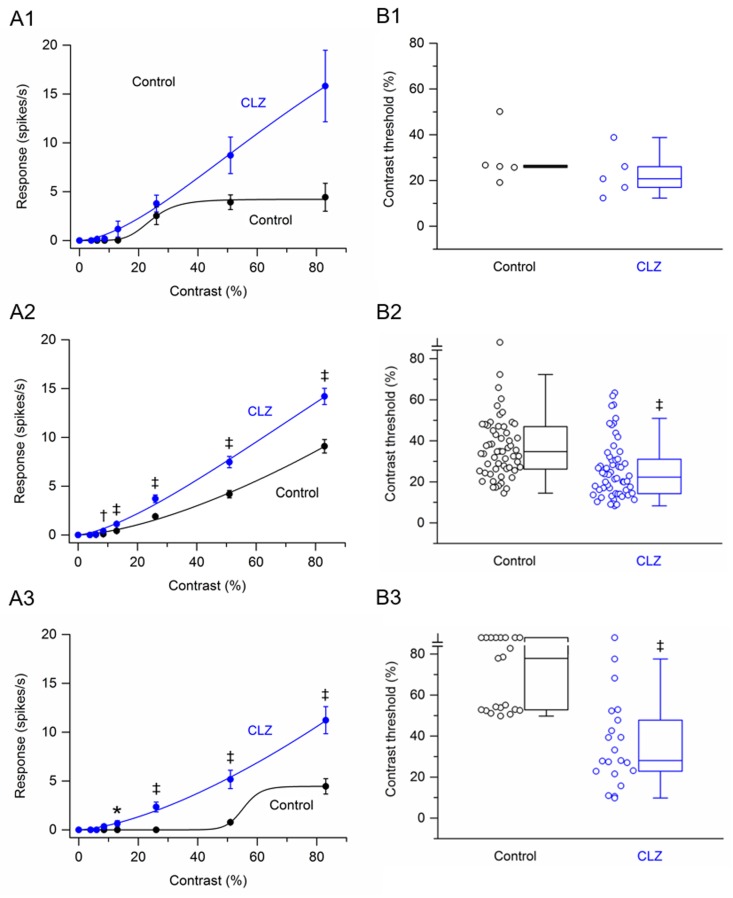
Effects of clozapine on responses of P23H rat RGCs to drifting sinusoidal grating of various contrasts. (A1 –A3) RGC contrast response functions. (A1) Contrast response function from saturating RGCs (n = 5) before and after application of clozapine. (A2) Contrast response function from non-saturating RGCs (n = 58) before and after application of clozapine. (A3) Contrast response function from uncategorized RGCs (n = 21) before and after application of clozapine. Data points in (A1), (A2) and (A3) are the mean ± SEM. * P < 0.05, † P < 0.01, ‡ P < 0.001 (Holm-Bonferroni multiple correction). Note that the control curve in (A3) is a bad fit, giving the impression that the cells were ‘saturating’ cells. This bad fit is because only two data points have values greater than zero. (B1 –B3) RGC contrast thresholds. (B1) Contrast thresholds for saturating RGCs (n = 5) before and after application of clozapine. (B2) Contrast thresholds for non-saturating RGCs (n = 58) before and after application of clozapine. (B3) Contrast thresholds for uncategorized RGCs (n = 21) before and after application of clozapine. In (B1), (B2) and (B3), boxes represent the interquartile range (IQR) between first and third quartiles and the line inside represents the median. Whiskers denote the lowest and highest values within 1.5 x IQR from the first and third quartiles. Circles represent all data points. ‡ P < 0.001 (Wilcoxon signed-rank test). Note the contrast threshold values for one cell in (B2) and nine cells in (B3) were immeasurable (i.e., exceeded 83%).

Data were obtained from 84 RGCs (2 retinas at 15 lux, n = 40 cells; 1 retina at 30 lux, n = 20 cells; 2 retinas at 60 lux, n = 24 cells) that were treated with the antipsychotic drug clozapine. Of these RGCs, only 5 cells were saturating cells. Fig 5(A1) shows the contrast response function averaged from these saturating RGCs before and after application of clozapine. Although the response amplitudes from these cells were on average larger in the presence of clozapine, the increases were not found to be statistically significant. Fig 5(A2) shows the contrast response function averaged from non-saturating RGCs (n = 58) before and after application of clozapine. Clozapine significantly increased the response amplitudes to gratings of contrast from 8.5% to 83%. The greatest percentage increase, 333%, occurred at 8.5% contrast. At higher contrasts (13% to 83%), clozapine increased the response amplitudes on average by 56–168%. Fig 5(A3) shows the contrast response function averaged from uncategorized RGCs (n = 21) before and after application of clozapine. Before application of clozapine, no responses were observed from any cell until the contrast reached 53%. In the presence of clozapine, 3 cells responded to 8.5% contrast, 5 cells responded to 13% contrast, and 17 cells responded to 26% contrast. Statistically, clozapine significantly increased the response amplitudes to gratings of contrast from 13% to 83%.

Contrast thresholds were determined for saturating, non-saturating, and uncategorized RGCs. The data are displayed as three box plots in (Fig 5B1, 5B2 and 5B3). For the population of saturating RGCs, the median contrast thresholds were 26.1% before application of clozapine and 20.8% after application of clozapine. The difference between the medians was not statistically significant (P = 0.625). For the population of non-saturating RGCs, the median contrast thresholds were 34.8% before application of clozapine and 22.3% after application of clozapine. The difference between the medians was statistically significant (P < 0.001). For the population of uncategorized RGCs, the median contrast thresholds were 77.9% before application of clozapine and 28.1% after application of clozapine. The difference between the medians was statistically significant (P < 0.001). Taken together, clozapine decreased the contrast thresholds of P23H rat RGCs.

## Discussion

In this study I examined the effects of the antipsychotic drugs haloperidol and clozapine on the responses of RGCs in both SD rats and P23H rats to a drifting sinusoidal grating of various contrasts. The effects of haloperidol and clozapine on the responses of the RGCs to the drifting grating are for the most part very similar. In general, the antipsychotic drugs 1) decrease response amplitudes of SD rat RGCs but increase response amplitudes of P23H rat RGCs, and 2) increase contrast thresholds of SD rat RGCs but decrease contrast thresholds in P23H rat RGCs. One striking difference between clozapine and haloperidol is that latter did not reduce the response amplitudes of non-saturating cells in SD rat retinas. Although both clozapine and haloperidol block dopamine D2 receptors, clozapine—unlike haloperidol—has a high affinity for serotonin (5-HT) receptors, particularly the 5-HT2 receptor subtype [21]. Given that the retina expresses 5-HT2 receptors [22, 23], the clozapine-induced decrease in the response amplitudes of non-saturating SD cells may be to the binding of clozapine to these receptors. Future studies in this area should be conducted with selective receptor antagonists.T 2 receptors……

The finding of differential effects of the antipsychotic drugs on the contrast response functions of SD and P23H rat RGCs is not without precedence. Similar findings were observed with the GABA_C_ receptor antagonist TPMPA and the mGlu1 receptor antagonist JNJ16259685 [16]. Both receptor antagonists increase the response amplitudes of saturating and non-saturating P23H rat RGCs to all grating contrasts, while either not affecting the response amplitudes of SD rat RGCs (in the case of JNJ16259685) or having mixed effects depending upon the grating contrast (in the case of TPMPA). The similarity of effects of TPMPA, JNJ16259685, clozapine and haloperidol on the response amplitudes and contrast thresholds of RGCs in P23H rat retinas suggests that these drugs are acting on a common, anatomical pathway in the retina. Could it be the primary rod pathway? The primary rod pathway includes rod bipolar cells and AII amacrine cells [24]. Both GABA_C_ receptors and 5-HT2 receptors are expressed in rod bipolar cells [22, 25], and AII amacrine cells receive synaptic input from dopaminergic amacrine cells [26], which are modulated by dopamine D2 receptor ligands [27, 28]. In animal models of RP, histological changes have been reported in both rod bipolar cells and AII amacrine cells [29–31]. Since AII amacrine cells provide a conduit for transmission of visual signals from rod bipolar cells to cone bipolar cells [32], a dysfunctional rod pathway could adversely impact cone pathways to RGCs [33]. Studying the effects of antipsychotic drugs, as well as GABA_C_ and mGlu1 receptor antagonists, on rod bipolar cells and AII amacrine cells in SD and P23H rat retinas may be worthwhile.

Is there any evidence that antipsychotic drugs affect contrast sensitivity in humans?

Antipsychotic drugs are commonly used to treat people diagnosed with schizophrenia.

Although many studies have documented changes in contrast sensitivity in schizophrenia subjects, it is difficult to assess the extent to which the changes are due to the diseased state or the antipsychotic medication. Two studies have addressed this issue by examining contrast sensitivity in unmedicated and medicated schizophrenia patients. Chen et al. [34] and Cadenhead et al. [35] both reported that compared to unmedicated schizophrenia patients medicated schizophrenia patients show lower contrast sensitivities. It will be of interest determine if antipsychotic drugs decrease contrast sensitivity in healthy individuals as well, as the findings reported here on healthy SD rat RGCs would suggest.

To conclude, the results presented here suggest that an antipsychotic drug may improve contrast sensitivity in patients with retinitis pigmentosa and possibly other retinal diseases in which there is photoreceptor degeneration with concomitant remodeling of cells within the inner retina.

## Supporting information

S1 Dataset This dataset contains the data points summarized in figures. Data for each figure are presented on separate sheets.
(XLSX)
